# Social Determinants of Health: An Evaluation of Risk Factors Associated With Inpatient Presentations in the United States

**DOI:** 10.7759/cureus.13287

**Published:** 2021-02-11

**Authors:** Saanie Sulley, Mathios Bayssie

**Affiliations:** 1 Health Informatics, National Healthy Start Association, Washington DC, USA; 2 Emergency Medicine, Southern Tennessee Regional Health System, Nashville, USA

**Keywords:** culture and social determinants of health, housing difficulties, healthcare inequality, mental health literacy, race inequities, health care disparities, mental health comorbidities, psycho-behavioral, unemployment, • access to healthcare and health outcomes of vulnerable populations

## Abstract

Social Determinants of Health (SDoH) are socioeconomic indicators that directly or indirectly impact individual and community health outcomes. The distribution of most of these indicators within communities can be traced to public policies. These public policies often lead to diverse inequities with varying impacts on communities across the country. The inequities that arise because of specific public policies can be associated with increased risk factors and poor health outcomes among communities at high risk for these indicators. This study examined inpatient hospitalization and SDoH indicators that put individuals at risk of poor health outcomes. We utilized the National Inpatient Sample (NIS) databases 2012-2014 and 2016-2017 through the Healthcare Cost and Utilization Project (HCUP). The NIS datasets are de-identified to ensure patients' privacy. The HCUP-NIS dataset is a well-established sizable all-payer inpatient dataset for national estimates. It includes primary, secondary inpatient diagnoses as well as demographic information. SDoH indicators were identified using the International Classification of Diseases (ICD), versions 9 and 10 diagnosis codes. The relationship between SDoH indicators such as housing, psychosocial, healthcare access, upbringing, unemployment, social factors, gender, race, income, region, payer, age group, mortality, and severity was evaluated in a regression analysis. A total of 3,002,557 (2012-2014) and 1,254,899 (2016-2017) cases were included in this study. Mental diseases (p < 0.001) were high between 2012-2014 (OR 18.8, 95% CI 18.20-19.42) and 2016-2017 (OR 4.11, 95% CI 3.99-4.23). Native Americans had odds of presentation (p < 0.001) with SDoH indicator between 2012-2014 (OR 1.15, 95% CI 1.12-1.18) and 2016-2017 (OR 1.75, 95% CI 1.70-1.79). The odds of presentation among long income group were high compared to other income categories (p < 0.001) between 2012-2014 (OR 1.15, 95% CI 1.15-1.16) and 2016-2017 (OR 1.26, 95% CI 1.28-1.32). In conclusion, disparities, severity, and mortality risk at presentation were high among minority communities, males, and low-income demographics across all regions of United States

## Introduction

Social Determinants of Health (SDoH) are essential indicators that directly or indirectly impact one's ability to effectively manage medical conditions and outcomes [1-3]. In recent years, there have been concerted efforts at the local and national levels to decrease the disparities that exist in healthcare outcomes through the healthy people initiative [4]. Given the variances in presentations, health outcomes among individuals meeting these SDoH classifications need careful evaluation to ensure practical and strategic approaches to addressing them [5,6]. These SDoH have been shown to disproportionally affect low-income and minority communities across the country [7-9]. These indicators could hold the key to identify the most vulnerable populations in communities and to target them for preventive interventions. Inpatient data is rarely utilized in understanding some of the factors that play a crucial role in patient presentations. Utilization of such data in combination with the outpatient and community service providers’ data could provide a much better overview of the impact of such determinants on hospitalization and community resource allocation. Furthermore, it can tailor needed care to these populations based on specific needs [10]. This study evaluates the dynamics associated with socioeconomic and demographic factors impacting inpatient presentation of patients with SDoH indicators.

These valuable markers have been inadequately used to better coordinate care and improve community health outcomes. This study aims to provide an overview of how healthcare organizations and hospitals or direct care providers could inculcate SDoH indicators as part of their community health evaluation and assessment [11]. The level of care coordination and collaboration of such determinants could be the thriving force to improve outcomes, especially among the most vulnerable populations in our communities. Understanding the unique characteristics associated with these presentations could help targeted approaches to policy and strategic accountability to ensure equitable care approaches in communities across the country.

## Materials and methods

This retrospective analysis of inpatient hospitalization with SDoH was conducted using National Inpatient Sample (NIS) databases 2012-2014 and 2016-2017 available through the Healthcare Cost and Utilization Project (HCUP). The Agency for Healthcare Research and Quality (AHRQ) sponsors the HCUP databases. The NIS datasets are de-identified to ensure patients' privacy; providers and hospitals are also de-identified in the NIS datasets.

HCUP-NIS is a well-established sizable all-payer inpatient dataset for national estimates. The dataset includes primary and secondary inpatient diagnoses as well as demographic information. SDoH presentations were included if coded as primary or secondary diagnosis using International Classification of Diseases (ICD) 9/10 CM codes (Table 1). The SDoH indicators included are housing difficulties, family circumstances, psychosocial issues, healthcare access, unemployment, education and literacy, problems related to upbringing, as well as social and environmental factors. These indicators correspond to ICD-9 (V60-60) and ICD-10 (Z59-Z75). A total of 3,002,558 and 1,254,899 SDoH cases were identified between 2012-2014 and 2016-2017 in the NIS dataset, respectively.

**Table 1:** Study Sample Characteristics and Inclusion Summary *Records with missing cases were excluded from the analysis. SDoH: Social Determinants of Health; ICD: International Classification of Diseases.

Study Sample Characteristics		
Variables of Interest	N	
Inclusion (N-weighted)	2012-2014 (%)	2016-2017 (%)
*Combined SDoH Indicator (Housing, Family, Psychosocial, Healthcare Access, Education, Unemployment, Upbringing, Social Environment)	3,002,557 (2.8)	1,254,899 (1.8)
Housing Difficulties	726,313 (20)	774,022 (55)
Family Circumstances	435,788 (12)	216,726 (14)
Psychosocial Issues	1,997,362 (55)	139,323 (9)
Healthcare Access	36,315 (1)	3,096 (0.2)
Unemployment	290,525 (8)	201,245 (13)
Education and Literacy	36,315 (1)	23,220 (1.5)
Upbringing	10,894 (0.3)	278,647 (18)
Social Environment	72631 (2)	201,240 (13)
Gender		
Female	1,416,364 (47)	526,315 (42)
Male	1,585,669 (53)	728,329 (58)
Race/Ethnicity		
White	1,844,269 (66)	745,684 (59)
Black	508,700 (18)	248,620 (20)
Hispanic	283,305 (10)	125,375 (10)
Asian or Pacific Islander	37,505 (1)	16,610 (1.3)
Native American	19,865 (0.7)	13,040 (1)
Other	89,130 (3)	34,805 (2.8)
Payer		
Medicare	779,120 (26)	312,160 (25)
Medicaid	995,989 (33)	565,764 (45)
Private Insurance	672,364 (22)	193,125 (15)
Self-Pay	334,815 (11)	112,910 (9)
No Charge	44,965 (2)	13,755 (1)
Other	167,905 (6)	52,645 (4)
Census Division		
New England	192,800 (6)	77,320 (6.2)
Middle Atlantic	448,420 (15)	168,870 (14)
East North Central	465,890 (16)	177,290 (14)
West North Central	266,049 (9)	95,375 (8)
South Atlantic	617,780 (21)	245,370 (20)
East South Central	159,530 (5)	67,560 (5)
West South Central	260,850 (9)	98,200 (8)
Mountain	157,843 (5)	89,925 (7)
Pacific	433,395 (14)	234,989 (19)
Household Income		
< $43,000	984,359 (35)	434,995 (38)
< $54,000	746,764 (26)	291,485 (26)
< $71,000	620,649 (22)	242,355 (21)
$71,000+	486,770 (17)	174,740 (15)
Age Group (Years)		
< 17	253,454 (9)	93,690 (8)
18-24	323,090 (11)	103,650 (8)
25-34	534,505 (18)	205,650 (16)
35-44	491,205 (17)	198,020 (16)
45-54	636,585 (21)	254,280 (20)
55-64	391,520 (13)	217,110 (17)
65+	354,510 (12)	168,620 (13)

These data were further analyzed by the most frequent Major Diagnostic Category (MDC) presentation in the selected SDoH presentations. The relationship between SDoH presentations by race, gender, hospital division, age, income, severity, mortality risk, payer, length of stay (LOS), number of diagnoses (NDX), and disposition was examined in the regression analysis. Discharge weight (provided in the NIS database) was applied to all calculations to attain a national representative sample. Weighted descriptive statistics, multivariate logistic regression, was conducted for the relationship between the variables.

We used Statistical Package for Social Sciences (SPSS) version 23.0 (IBM Corp., Armonk, NY) to conduct a retrospective analysis of the HCUP-NIS database from 2012 to 2014 and 2016 to 2017. Records with missing data were excluded from all calculations. We also used Microsoft Excel (Microsoft Corporation, Redmond, WA) to calculate the rates and graph the statistics in this study. The 2015 NIS data were not included because of the United States switch to ICD-10 in 2015.

## Results

**Figure 1:**
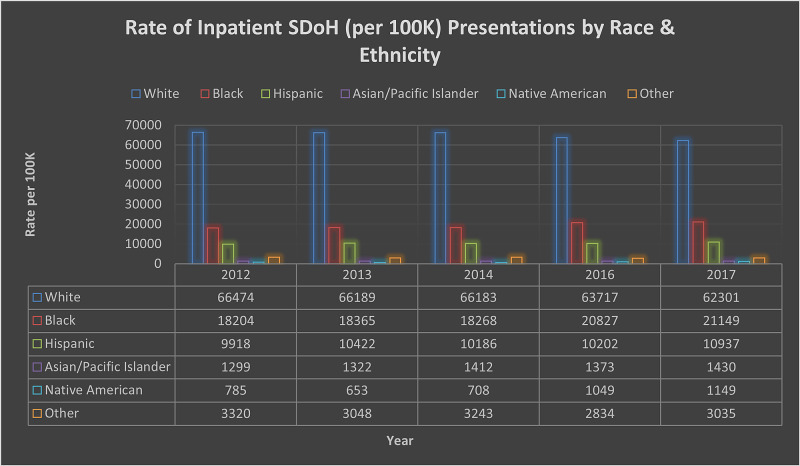
Rate of Inpatient SDoH (per 100K) Presentation by Race and Ethnicity SDoH: Social Determinants of Health.

A total of 3,002,557 (2012-2014) and 1,254,899 (2016-2017) cases with combined primary and secondary SDoH indicators were identified. Figure 1 shows a variation in the presentation by race when compared with the ICD-9/10. This variation could be associated with a lack of knowledge or utilization of SDoH codes. Table 1 provides the descriptive statistics and analysis for the varied demographic characteristics of the study sample.

The odds of mental disease and disorders presentation were high between 2012-2014 (OR 18.8, 95% CI 18.20-19.42) and 2016-2017 (OR 4.11, 95% CI 3.99-4.23). The variation in presentation could be associated with several factors, including the utilization of codes. The relationship between SDoH indicator and alcohol and the drug-related presentation were significant (p < 0.001) between 2012-2014 (OR 5.02, 95% CI 4.86-5.19) and 2016-2017 (OR 2.10, 95% CI 2.04-2.16).

The population's racial and ethnic demographics show significance among all classification with individuals identified as White with OR 1.19 (95% CI 1.18-1.20) between 2012 and 2014 and Other with OR 1.35 (95% CI 1.28-1.32) between 2016 and 2017. This finding shows a variation in the patient population's presentation as shown in Figure 1 and Table 2. The likelihood of presentation among males varied slightly with OR 1.17 (95% CI 1.17-1.18) and OR 1.30 (95% CI 1.29-1.30) between 2012-2014 and 2016-2017. Both presentations were statistically significant (p < 0.001).

**Table 2:** Logistic Regression Analysis for SDoH Cases (2012-2014 and 2016-2017) *Reference. SDoH: Social Determinants of Health.

	2012-2014		2016-2017		P value (Sig)	
Variables	OR	95 CI	OR	95 CI	2012-2014	2016-2017
Disposition						
Routine Discharge	0.88	0.81-0.95	1.57	1.23-2.01	0.002	<0.001
Transfer to Short-term Hosp	0.79	0.73-0.85	1.44	1.13-1.84	0.003	0.003
Transfer Other: Includes Skilled Nursing Facility	1.07	0.99-1.15	1.76	1.38-2.25	0.070	0.001
Home Health Care (HHC)	0.57	0.53-0.62	0.92	0.72-1.18	<0.001	0.535
Against Medical Advice	1.17	1.08-1.26	2.8	2.21-3.61	<0.001	<0.001
*Discharged Alive						<0.001
Most Common Diagnoses (Major Diagnostic Category)						
Mental Disease and Disorders	18.80	18.2-19.4	4.11	3.99-4.23	<0.001	<0.001
Alcohol and Drug Use	5.02	4.86-5.19	2.04	2.04-2.16	<0.001	<0.001
Injuries, Poisonings, and Toxic Effects of Drugs	1.68	1.62-1.73	0.79	0.76-0.81	<0.001	<0.001
Sex						
*Female						
Male	1.17	1.7-1.18	1.30	1.29-1.30	<0.001	<0.001
Race						
*Other						<0.001
White	1.19	1.18-1.20	1.30	1.28-1.32	<0.001	<0.001
Black	1.04	1.03-1.05	1.26	1.25-1.28	<0.001	<0.001
Hispanic	0.93	0.92-0.94	0.86	0.85-0.87	<0.001	<0.001
Asian or Pacific Islander	0.72	0.70-0.72	0.71	0.70-0.73	<0.001	<0.001
Native American	1.19	1.12-1.21	1.75	1.70-1.79	<0.001	<0.001
Census Region						
New England	0.94	0.93-1.94	1.02	1.01-1.03	<0.001	<0.001
Middle Atlantic	0.59	0.59-0.60	0.64	0.63-0.64	<0.001	<0.001
East North Central	0.69	0.68-0.69	0.66	0.66-0.67	<0.001	<0.001
West North Central	0.78	0.77-0.79	0.65	0.65-0.66	0.126	<0.001
South Atlantic	0.73	0.72-0.74	0.68	0.68-0.69	<0.001	<0.001
East South Central	0.63	0.62-0.64	0.57	0.57-0.58	<0.001	<0.001
West South Central	0.65	0.65-0.66	0.59	0.59-0.60	<0.001	<0.001
Mountain	0.80	0.80-0.81	0.95	0.94-0.96	<0.001	<0.001
*Pacific					<0.001	<0.001
Primary Payer						
Medicare	0.53	0.53-0.54	0.52	0.51-0.52	<0.001	<0.001
Medicaid	1.13	1.12-1.14	1.34	1.30-1.32	<0.001	<0.001
Private Insurance	0.70	0.69-0.71	0.51	0.50-0.51	<0.001	<0.001
Self-Pay	1.37	1.36-1.39	1.47	1.46-1.49	<0.001	<0.001
No Charge	1.16	1.58-1.64	1.79	1.75-1.83	<0.001	<0.001
*Other					<0.001	<0.001
Rural-Urban Code						
"Central" Counties of Metro Areas of ≥1 Million Population	1.30	1.29-1.31	1.41	1.40-1.42	<0.001	<0.001
"Fringe" Counties of Metro Areas of ≥1 Million Population	1.19	1.18-1.20	1.21	1.20-1.23	<0.001	<0.001
Counties in Metro Areas of 250,000-999,999 Population	1.32	1.30-1.33	1.35	1.33-1.36	<0.001	<0.001
Counties in Metro Areas of 50,000-249,999 Population	1.31	1.30-1.33	1.37	1.35-1.38	<0.001	<0.001
Micropolitan Counties	1.20	1.95-1.21	1.11	1.09-1.12	<0.001	<0.001
*Not Metropolitan or Micropolitan Counties					<0.001	0.005
Median Household Income						
< $43,000	1.15	1.15-1.16	1.26	1.28-1.32	<0.001	
< $54,000	1.07	1.06-1.08	1.10	1.10-1.11	<0.001	0.001
< $71,000	1.02	1.02-1.03	1.06	1.05-1.06	<0.001	<0.001
*$71,000+					<0.001	<0.001
Severity Risk						
Minor Loss of Function (Includes Cases With No Comorbidity or Complications)	0.64	0.63-0.65	0.54	0.54-0.55	0.001	<0.001
Moderate Loss of Function	0.86	0.85-0.87	0.90	0.89-0.92	<0.001	<0.001
Major Loss of Function	1.00	0.98-1.01	1.15	1.13-1.16	0.867	<0.001
*Extreme Loss of Function					<0.001	<0.001
Mortality Risk (%)						
*Did Not Die						
Died	1.75	1.61-1.89	1.09	0.85-1.39	<0.001	0.500
No Class Specified	0.69	0.55-0.88	1.67	1.41-1.99	<0.003	<0.001
Minor Likelihood of Dying	1.53	1.50-1.55	1.80	1.77-1.83	<0.001	<0.001
Moderate Likelihood of Dying	1.53	1.51-1.56	1.49	1.46-1.53	<0.001	<0.001
Major Likelihood of Dying	1.14	1.12-1.15	1.13	1.11-1.15	<0.001	<0.001
*Extreme Likelihood of Dying					<0.001	<0.001

Emergency department (ED) presentations were significant (p < 0.001) for SDoH indicators with OR 1.10 (95% CI 1.09-1.11) between 2012 and 2014 and OR 0.91(95% CI 0.90-0.92) between 2016 and 2017. A significant relationship was observed in all regions with an increased odd of presentation in New England between 2012-2014 (OR 0.94, 95% CI 0.93-0.94) and 2016-2017 (OR 1.02, 95% CI 1.01-1.04). The mortality rate among individuals with SDoH indicator was significant (p < 0.001) with an OR of 1.75 (95% CI 1.61-1.89) from 2012 to 2014.

**Figure 2:**
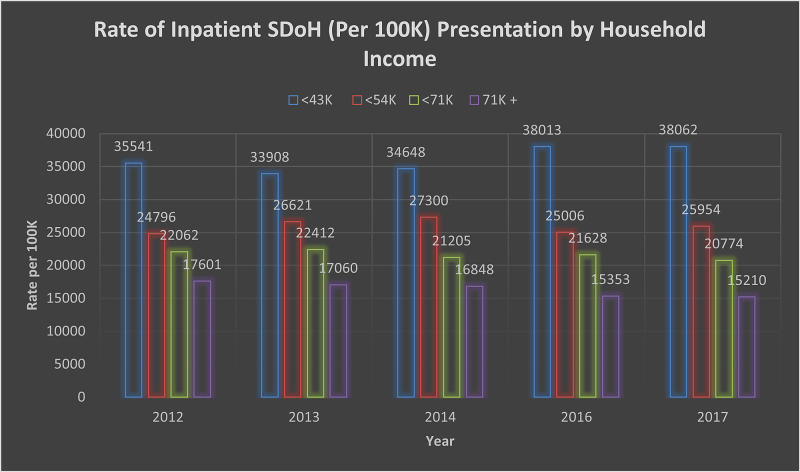
Rate of Inpatient SDoH (per 100K) Presentation by Household Income SDoH: Social Determinants of Health.

**Figure 3:**
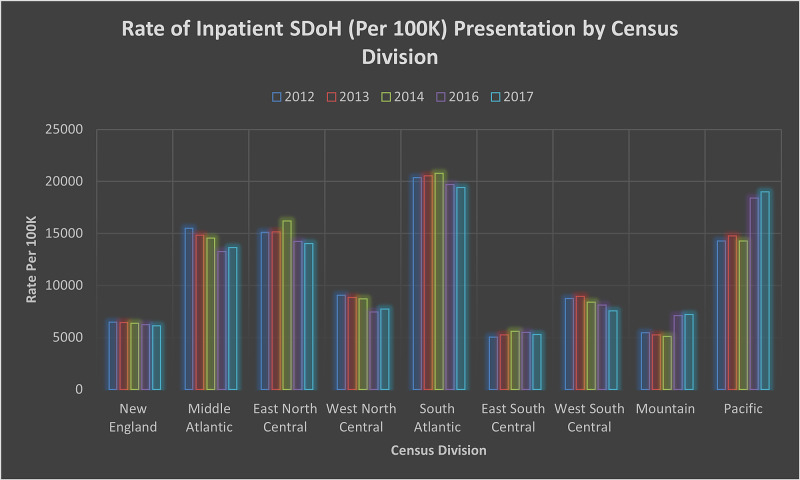
Rate of Inpatient SDoH (Per 100K) Presentation by Census Division SDoH: Social Determinants of Health. New England: Maine, New Hampshire, Vermont, Massachusetts, Rhode Island, and Connecticut. Mid-Atlantic: New York, Pennsylvania, and New Jersey. East North Central: Wisconsin, Michigan, Illinois, Indiana, and Ohio. West North Central: Missouri, North Dakota, South Dakota, Nebraska, Kansas, Minnesota, and Iowa. South Atlantic: Delaware, Maryland, District of Columbia, Virginia, West Virginia, North Carolina, South Carolina, Georgia, and Florida. East South Central: Kentucky, Tennessee, Mississippi, and Alabama. West South Central: Oklahoma, Texas, Arkansas, and Louisiana. Mountain: Idaho, Montana, Wyoming, Nevada, Utah, Colorado, Arizona, and New Mexico. Pacific: Alaska, Washington, Oregon, California, and Hawaii.

Further analysis shows a significant relationship between patient's discharge against medical advice and the presence of most of the SDoH indicators included in this study. Payer demographics shows significance (p < 0.001) among individuals without insurance (No Charge) between 2012-2014 (OR 1.61, 95% CI 1.58-1.64) and 2016-2017 (OR 1.79, 95% CI 1.75-1.83). A significant presentation was observed across the country based on rural-urban classification, as indicated in Table 2. The odds of presentation based on the lowest income group were statistically significant with p < 0.001 between 2012-2014 (OR 1.16, 95% CI 1.15-1.16) and 2016-2017 (OR 1.26, 95% CI 1.25-1.27). Figure 2 provides an overview of income demographics in the population. Inpatient presentation by region (Figure 3) with the North East among SDoH cases was significant, p < 0.001 (OR 1.02, 95% CI 1.01-1.03).

## Discussion

The SDoH presentation trend in each subcategory shows an increase when comparing ICD-9 (2012-2014) and ICD-10 (2016-2017). The reduction in actual case presentation may be associated with the switch from ICD-9 to ICD-10 and more specificity in the classification of these determinants, as shown in Figure 1. In recent years, efforts to capture SDoH in EHR systems have increased and could also be one of the many reasons for less utilization of the ICD codes. Psychosocial, housing difficulties, and unemployment make up the highest percentage in presentation among the population. These findings are consistent with other published literature regarding the prevalence of social determinants of health [12-14].

This study shows consistent high odds of presentation for mental and behavioral health among this population. This further highlights the need for more focus and investment in mental and behavioral health to mitigate disproportionate health outcomes among these populations [15-18]. Such investment in mental and behavioral health is even more timely, given the increase in the rate of SDoH in all communities across the country because of the COVID-19 pandemic. The current approaches to care need to be re-evaluated [19,20] to focus more on co-operation [18] and to create an effective referral system to ensure populations needing social services have access to such services in tandem with their medical needs. Community-based mental health services and approaches also need to be adopted and implemented to improve health outcomes [21].

This study demonstrates that Native Americans are consistently at higher odds of having SDoH classification than other race or ethnic demographics in the United States. The significant severity and mortality risk at presentation was also consistently high among Native Americans, Asian/Pacific Islander, and Black between 2012-2014 and 2016-2017. It shows that the increased risk of disease severity and mortality in minority communities is associated with a higher risk of negative SDoH. A review of severity and mortality risks at a presentation by household income as a SDoH indicator shows consistently higher moderate risks among lower income and many minority populations between 2012-2014 and 2016-2017. Such findings are consistent with other studies that have found higher stressors among low-income and minority communities [16,22-25]. These findings call for a re-evaluation of public policy on social and economic inequalities and support models focusing on minority populations to mitigate poor SDoH.

The highest percentage age distribution at presentation was 45-54 years (20%); 12.5% of the study population in the data were above 65 years of age. The number of older adults meeting these criteria seems to be increasing, as shown in Table 1. As age increased, the risk of significant severity and mortality at presentation increased across all country census divisions. The consistent utilization rate of Medicaid and Medicare as primary coverage further supports the need for a multistakeholder approach to addressing SDoH in communities, especially among the elderly living in communities. This finding also shows that local governments and policymakers are an indispensable part of a strategy for addressing social factors impacting health outcomes [14, 26]. Decisions on resource allocation, job programs, subsidized/affordable housing can improve health outcomes, and policymakers are at the forefront of implementing such changes affecting SDoH.

Development and implementation of community engagement strategies focused on addressing these social factors impacting health could pave the way for improving health outcomes. Tailoring programs based on community needs, evidence, and key performance indicators could help develop public, private, and community collaboratives to address social factors impacting health outcomes. The use of ICD codes alone may not be the most effective strategy in understanding these presentations' social dynamics. A combination of ICD codes and EHR data could provide a better understanding of these presentations. It could also serve as an avenue to improve the quality of data collected in an inpatient setting. The use of technology in referral processes could also hold the key to collaboration between inpatient, outpatient, and social service providers. Studies have found that even though there is an increase in health system investment in areas associated with improving SDoH, these investments only make up a small fraction of total healthcare expenditure [27,28]. Given the transient nature of some of these SDoH [29], it is imperative to address them effectively to limit exacerbating medical conditions and worsening population health indicators. Collaboration between all service providers is essential to manage SDoH issues affecting health outcomes in diverse communities.

This retrospective study cannot conclude causation. It provides an overview of SDoH code utilization/presentation in inpatient settings across the United States between 2012-2014 (ICD-9) and 2016-2017 (ICD-10). It further provides insights into how inpatient service providers could inculcate SDoH codes in developing comprehensive community health needs assessments. Such an approach could help all community stakeholders develop adequate data-driven policy and implementation strategies to improve health outcomes among individuals with SDoH risk factors.

Limitations

The utilization of multiple versions of ICD may have impacted the inclusion criteria. The use and adoption of new codes, especially when they are not reimbursable, may have affected the number of cases for both ICD-9 and ICD-10. Increasing the use of other EHR system features could be a limiting factor in capturing SDoH through ICD code. A combination of ICD codes and EHR data in analyzing SDoH would provide a more comprehensive overview of these indicators.

## Conclusions

SDoH are significant risk factors for lowered health outcomes for minority populations. Many SDoH can be addressed during outpatient visits with practical preventive care approaches tailored toward addressing these risk factors based on local community dynamics. High risk among low-income earners, minority racial groups, and public payer groups in most regions indicates a need for focused collaborative approaches to improving health outcomes through active community-based partnerships to address social needs.

## Appendices

**Table 3:** ICD-9/10 Codes Inclusion Criteria ICD: International Classification of Diseases; SDoH: Social Determinants of Health.

SDoH Indicator	ICD-9 Code	ICD-10 Code
Housing Difficulties	V60.XX	Z59.XX
Family Circumstances	V61.03, V61.49, V61.8, V61.9	Z63.XX
Psychosocial Issues	V61.5, V61.6, V61.7, V62.81	Z64.XX
Healthcare Access	V63.8, V63.9	Z75.X
Unemployment	V62.XX	Z56.XX
Education and Literacy	V62.3	Z55.XX
Upbringing	V61.XX	Z62.XX
Social Environment	V62.89, V60.3, V62.4	Z60.XX
